# Disturbance of lipid metabolism in germ-free mice transplanted with gut microbiota of DSS-induced colitis mice

**DOI:** 10.1371/journal.pone.0280850

**Published:** 2023-02-03

**Authors:** Chungho Lee, SangAh Kim, Bobae Kim, Wilhelm H. Holzapfel, Chang-Kee Hyun

**Affiliations:** 1 School of Life Science, Handong Global University, Pohang, Gyungbuk, Republic of Korea; 2 Basic Research Center, HEM Pharma Inc., Pohang, Gyungbuk, Republic of Korea; 3 Department of Advanced Convergence, Handong Global University, Pohang, Gyungbuk, Republic of Korea; Tokyo University of Agriculture, JAPAN

## Abstract

Hepatobiliary abnormality and metabolic disorders are frequently observed complications in patients with inflammatory bowel diseases (IBD). Given that microbiota dysbiosis is a common pathophysiological feature of both IBD and metabolic diseases, we examined how the IBD-induced dysbiosis affects the host metabolism and contributes to the development of associated metabolic diseases using germ-free (GF) mice transplanted with fecal microbiota of DSS-induced colitis mice. There was no significant change in inflammation or barrier integrity in the gut of GF mice that received microbiota from colitis mice compared to their counterparts that were transplanted with microbiota from non-colitis healthy mice. Interestingly, it was observed that the GF recipients of colitis-induced altered microbiota showed a significant decrease in the weight of adipose tissues including mesenteric, epididymal, subcutaneous, and brown fat without any change in body weight, which was accompanied by abnormalities in adipose tissue functions such as fat storage and adiponectin production. Transplantation of colitis-induced altered microbiota also disrupted hepatic lipid metabolism in the GF recipient mice, which was observed by increases in synthesis and accumulation of cholesterol and bile acids in hepatocytes and a decrease in plasma HDL-cholesterol. Additional observations including elevated plasma levels of insulin, decreased hepatic production of FGF21, and decreased levels of fecal short chain fatty acids (SCFAs) and hepatic expression of SCFA receptors led to a conclusion that the transplantation of the colitis-associated dysbiotic microbiota was causally associated with impairments of insulin action and FGF21-adiponectin axis, possibly due to the low SCFA-producing capacity of the colonized microbiota, leading to metabolic abnormalities including adipose tissue dysfunction and dysregulated hepatic lipid metabolism. Our findings suggest potential mechanisms that explain how colitis-associated gut dysbiosis may contribute to the development of metabolic dysfunctions, which could be applied to clinical practice to improve the efficacy of treatment of IBD patients with comorbid metabolic disorders or vice versa.

## Introduction

Inflammatory bowel disease (IBD), an umbrella term that refers to both ulcerative colitis (UC) and Crohn’s disease (CD), are multifactorial diseases characterized by chronic inflammation of all or part of the digestive tract. Although the etiology of IBD still remains unknown, there is a consensus that genetic susceptibility, environmental factors, dysregulated microbiota, and an excessive immune response are involved in the pathogenesis [1]. Besides the general symptoms of IBD such as persistent diarrhea, abdominal pain, bloody stools, and weight loss, there can be some complications in organs other than those of the gastrointestinal tract, which are termed extraintestinal manifestations [2]. Among those, liver and biliary tract disorders are typical extraintestinal manifestations in both UC and CD, and hepatic steatosis and primary sclerosing cholangitis (PSC) are common and have attracted special attention [3]. Several studies on the prevalence of hepatobiliary abnormalities in patients with IBD have reported that hepatic enlargement and steatosis were found in both UC and CD patients at higher prevalence rates compared to healthy controls [4,5].

In recent years, the gut microbiota and their metabolites have emerged as critical contributors to the control of host metabolism and immune functions [6]. Alterations in the gut microbiota and consequently dysregulated interaction with gut immunity have been known to be associated with the development of chronic inflammatory diseases such as metabolic disorders and IBD [7,8]. In addition, many recent studies have pointed out the role of disturbed metabolite profile derived from the altered gut microbiota in the pathogenesis of IBD, which also contributes to the pathogenesis of metabolic disorders [9]. Multi-omics studies on gut microbiota dysbiosis in patients with IBD have revealed a decreased abundance of producers of short-chain fatty acids (SCFAs), confirming the association of lowered levels of SCFAs with increased risk of IBD [10,11]. Particularly, the reduced butyrate-synthetic capacity of the microbiota has been revealed as a hallmark of IBD in many studies on IBD-associated gut dysbiosis [12–14]. Major SCFAs produced by gut microbiota are acetate, propionate and butyrate, which bind to G protein-coupled receptors (GPCRs) such as GPR41, GPR43 and GPR109a, and activate signaling cascades that can modulate the inflammatory response and increase the intestinal barrier integrity by enhancing the tight junction proteins functions [15]. Elevated acetate and butyrate have been found to stimulate GPR43 and GPR109a signaling to activate IL-18, promoting epithelial repair and protection against DSS-induced colitis [16]. Activation of GPR109a, which is the major receptor for butyrate, has been shown to lower the serum level of low-density lipoprotein (LDL)-cholesterol, whereas raise that of high-density lipoprotein (HDL)-cholesterol, reducing the risk of mortality from cardiovascular disease [17]. It has also been demonstrated to not only mediate an inhibition of lipolysis in adipocytes, but also upregulate the expression of ATP-binding cassette transporter A1 (ABCA1) leading to enhanced cholesterol efflux and HDL biosynthesis [18,19]. Although there is considerable epidemiological evidence indicating comorbidity between IBD and metabolic disorders, especially non-alcoholic fatty liver disease (NAFLD), the relationship between their molecular-level pathogenesis remains to be elucidated [20,21]. In our preceding study, we observed that DSS-induced colitis model mice developed NAFLD-like phenotypes such as hepatic steatosis and dyslipidemia, and found that the increased intestinal permeability and consequent endotoxemia caused by colitis were associated with hepatic inflammation and adipose tissue dysfunctions, thereby disrupting hepatic lipid and bile acid metabolism leading to excessive hepatic fat accumulation and abnormal lipid profile [22]. Given that the risk factors shared by IBD and NAFLD include intestinal barrier dysfunction and alterations in gut microbiota and their metabolites, here in this study, we hypothesized that the IBD-associated altered gut microbiota-derived metabolites may negatively affect host energy metabolism through crosstalk between metabolically active organs such as the liver, adipose, and skeletal muscle. To investigate the impact of colitis-associated gut dysbiosis on glucose and lipid metabolism in those tissues, we transplanted fecal microbiota from DSS-induced colitis mice and their non-colitis healthy controls into germ-free (GF) mice. GF mice transplanted with fecal microbiota of DSS-induced colitis mice showed a significantly reduced weight of adipose tissues and a disrupted lipid profile, which was associated with a modest increase in the liver weight, compared to their counterparts that were transplanted with non-colitis mice microbiota. Following these observations, we examined the molecular mechanism how IBD-associated gut dysbiosis contributes to the disturbance of lipid metabolism and the development of dysfunctions in adipose tissue and the liver.

## Materials and methods

### Animal experiments and fecal microbiota transplantation

**Fig 1:**
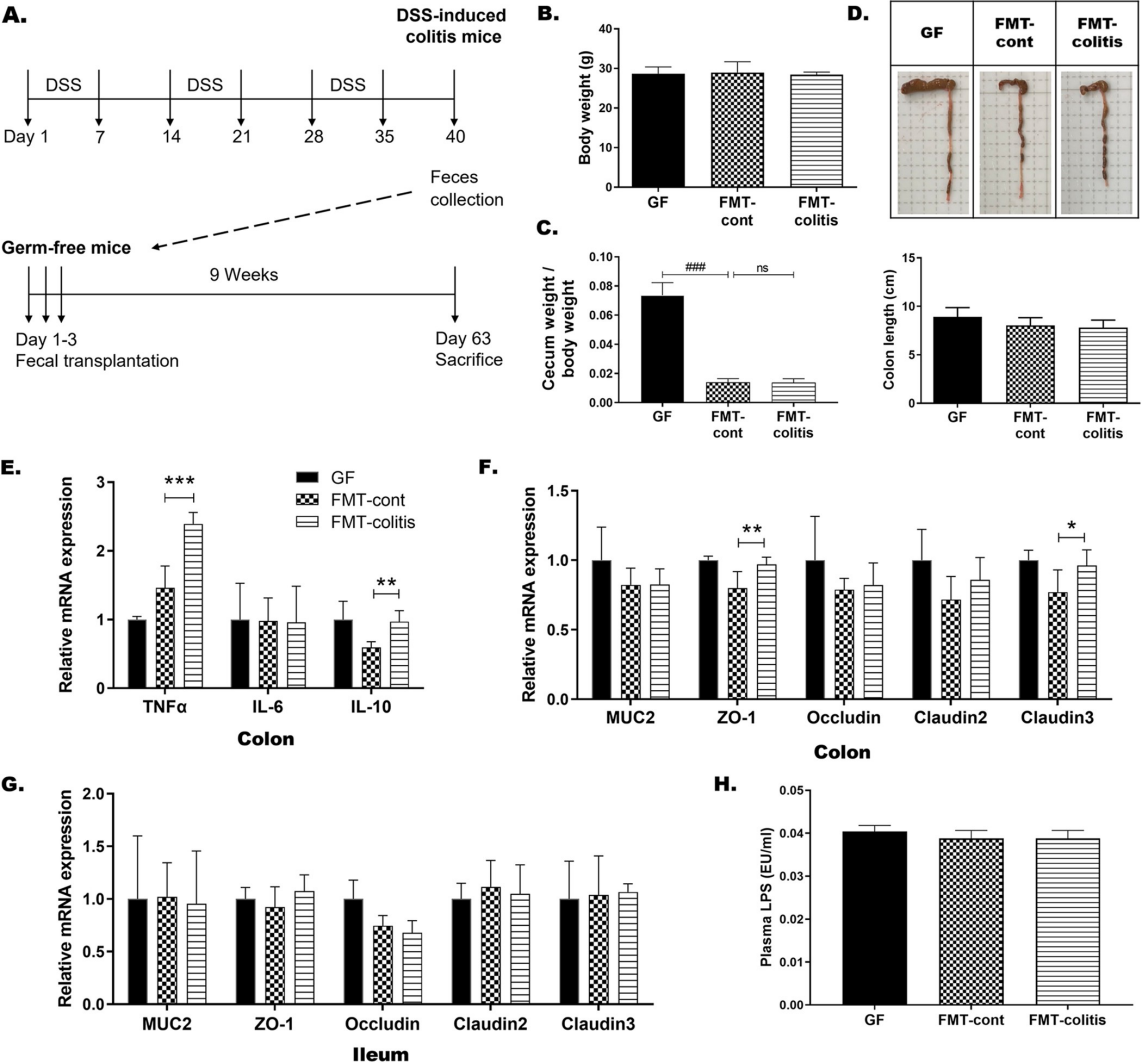
Transplantation of fecal microbiota from DSS-induced colitis mice did not drive inflammation or barrier disruption in the gut of GF recipient mice. (A) Experimental schedule. (B) Body weights and (C) cecum-to-body weight ratio at the time of sacrifice in each group of mice. (D) Representative photographs of colon and cecum from each group and a graph presenting the colon length. In B-D, statistical significance was determined by ordinary one-way ANOVA with Dunnett’s multiple comparison test (^#^*p* < 0.033, ^##^*p* < 0.002, and ^###^*p* < 0.001). The mRNA expression of (E) TNFα, IL-6, and IL-10 in colon, and (F and G) mucin and tight junction related genes in colon and ileum. (H) Plasma LPS levels measured by ELISA. Data are presented as the mean ± SD of each group (n = 5–7). In E-H, statistically significant difference between FMT-control and FMT-colitis group was determined by Student’s two-tailed t-test (*p < 0.05, **p < 0.01, and ***p < 0.001). GF: GF mice without fecal transplantation, FMT-cont: GF mice transplanted with fecal microbiota from non-colitis mice. FMT-colitis: GF mice transplanted with fecal microbiota from DSS-induced colitis mice.

Germ-free (GF) C57BL/6 mice, originally purchased from CLEA Japan Inc. (Tokyo, Japan) were bred and maintained in the gnotobiotic facility of HEM Pharma Inc. (Pohang, Korea) in sterile isolator under a 12-h light/dark cycle at 21 ± 1°C and 45 ± 10% humidity. All mice were given ad libitum access to standard chow (Teklad Global 18% Protein Rodent Diet, Teklad 2018s, Envigo, Madison, WI, USA), which was autoclaved and sterility tested prior to use. The inoculum for fecal microbiota transplantation (FMT) was prepared by sterile PBS dilution at 1:20 (w/v) of feces collected from chronic DSS-induced colitis mice, which were described in detail in our previous study [22]. FMT was performed as described previously [23]. Briefly, seven-week-old male GF mice were transferred to isolated cages (IsoCage P—Bioexclusion System, TECNIPLAST S.p.A., Buguggiate, Italy) and administered by oral gavage with 200 μL of the inoculum once a day for three consecutive days (Fig 1A). After 9 weeks of FMT, mice were sacrificed and the tissues including blood, liver, subcutaneous adipose tissue (SAT), epididymal adipose tissue (EAT), mesenteric adipose tissue (MAT), brown adipose tissue (BAT), soleus skeletal muscle, spleen, colon, and small intestine were collected, snap-frozen in liquid nitrogen, and kept at -80°C until processed for further analysis. The stool consistency and the rectal bleeding were assessed as described in our previous report [22]. Stool consistency was determined as follows: score 0, normal (solid pellet); 1, soft but in pellet shape; 2, loose stool but with some solidity; 3, loose stool with signs of liquid consistency; 4, watery diarrhea. Rectal bleeding was scored as follows: score 0, no sign of blood; score 1, no bleeding; 2, slight bleeding; 3, bloody diarrhea; 4, gross bleeding. All the experiments were performed in accordance with methods approved by the Committee on the Ethics of Animal Experiments of Handong Global University (Permit number: HGUIACUC20200420-006).

### Plasma analyses

To collect blood plasma, fresh blood was obtained via cardiac puncture and collected in a blood collection tube (BD Microtainer PST tube, BD Scientific, Franklin Lakes, NJ, USA), and subsequently centrifuged at 15,000 *g* for 5 min at room temperature. Plasma lipopolysaccharide (LPS) level was measured using ToxinSensor Chromogenic LAL Endotoxin Assay Kit (GenScript, Piscataway, NJ, USA). Plasma triglyceride (TG), total cholesterol, HDL cholesterol, and LDL cholesterol were measured using an automated analyzer, Mindray BS-390 (Mindray Bio-Medical Electronics Co., Nanshan, Shenzhen, China). Plasma levels of insulin and adiponectin were measured using a mouse insulin ELISA kit (ALPCO, Salem, NH, USA) and a mouse adiponectin/Acrp30 Quantikine ELISA kit (R&D systems, Minneapolis, MN, USA), respectively.

### Histological analysis

Tissue samples of the liver and adipose tissues were fixed in 10% (v/v) neutral buffered formalin. After paraffin blocks were made, sectioned with 5-μm-thick microtome and stained with hematoxylin and eosin (H&E). The sectioned samples were examined by EVOSTM M5000 Imaging system (ThermoFisher Scientific, Waltham, MA, USA) at x 200 magnification. Adipocyte size was calculated using ImageJ Adiposoft software [24]. To score the extent of discoloration of the liver tissue, the color intensity of images of the liver was analyzed using the open-source Fiji software [25]. The intensity of darkish grey-brown color was determined using a scale from 0 to 255 by obtaining the average gray value within each selected boxed area, which is the sum of the measured gray values of all the pixels divided by the number of pixels in the area.

### Hepatic lipid analyses

The level of TG and total cholesterol in the liver were measured by colorimetric assay using the TG Assay kit and the Total Cholesterol Assay kit (Asan Pharmaceutical, Seoul, Korea), respectively, as described previously [22].

### Real time RT-PCR

Quantitative real time RT-PCR was performed as described previously [22]. Briefly, total mRNA was extracted from homogenized tissues using Trizol reagent (Invitrogen, Waltham, MA, USA) and treated with DNase Ⅰ (Invitrogen) to remove genomic DNA contamination. Purified mRNA (1 μg) was used to synthesize complementary DNA using GoScript Reverse Transcriptase (Promega, Madison, WI, USA). Quantification of gene transcripts for acidic ribosomal phosphoprotein (Arbp), β-actin, glyceraldehyde-3-phosphate dehydrogenase (GAPDH), ATP-binding cassette transporter A1 (ABCA1), ATP-binding cassette subfamily G member 5 (ABCG5), ABCG8, acetyl-CoA carboxylase (ACC), acyl-CoA oxidase 1 (Acox1), apolipoprotein A1 (ApoA1), ApoB100, adipose triglyceride lipase (ATGL), bile salt export pump (BSEP), carbohydrate response element binding protein (ChREBP), Claludin2, Claudin3, carnitine palmitoyltransferase 1 (CPT1), cholesterol 7 alpha-hydroxylase (CYP7A1), CYP27A1, diacylglycerol acyltransferase 1 (DGAT1), DGAT2, fatty acid synthase (FAS), fibroblast growth factor 15 (FGF15), FGF21, farnesoid X receptor (FXR), fatty acid transport protein 2 (FATP2), FATP3, glycerol-3-phosphate acyltransferase (GPAT), GPR41, GPR43, GRP109a, HMG-CoA reductase (HMGCR), HMG-CoA synthase (HMGCS), hormone-sensitive lipase (HSL), interleukin-1β (IL-1β), IL-6, IL-10, low density lipoprotein receptor (LDLR), lipoprotein lipase (LPL), liver X receptor α (LXRα), medium-chain acyl-coenzyme A dehydrogenase (MCAD), monoacylglycerol lipase (MGL), monocyte chemoattractant protein-1 (MCP-1), mucin 2 (MUC2), occludin, peroxisome proliferator-activated receptor gamma coactivator-1 alpha (PGC1a), peroxisome proliferator-activated receptor α (PPARα), PPARγ, stearoyl-CoA desaturase 1 (SCD1), sterol-regulatory element binding protein 1c (SREBP1c), SREBP2, scavenger receptor class B type 1 (SR-B1), TGR5, tumor necrosis factor α (TNFα), and ZO-1 was performed using gene-specific forward and reverse primers and the relative expression levels of each gene were calculated using the ΔΔ Ct method and normalized to the expression of Arbp or GAPDH. Sequences of all primers used in this study are available in the S1 Table.

### Western blot analysis

Western blotting was performed as described previously [26]. Antibodies against adiponectin (Bioss Antibody, Woburn, MA, USA), FGF21 (Abcam, Cambridge, UK), HSP (Cell Signaling Technology [CST], Danvers, MA, USA), phospho-HSL (Ser563), β-actin (CST) and GAPDH (CST) were used as primary antibody, followed by anti-rabbit IgG-HRP conjugated secondary antibody (CST).

### SCFA analysis

Fecal concentrations of SCFAs were measured by means of gas chromatography coupled with a flame-ionization detector (GC-FID) as described by Zhang et al. [27]. Briefly, to extract SCFAs, 0.1 g fecal sample was vortexed in 1 mL distilled water and centrifuged. The supernatant of the centrifuged sample (150 μL) was transferred to a screw cap vial with 150 μL of GC buffer solution, which contains (NH_4_)_2_SO_4_, NaH_2_PO_4_, and 2-ethylbutric acid. GC-FID analysis was performed by Agilent 7890B GC system equipped with a 7697A headspace sampler and FID (Agilent Technologies, Wilmington, DE, USA). An HP-innowax capillary column (30 m x 0.32 mm i.d. x 0.50 μL film thickness; Agilent Technologies) was used with constant flow of nitrogen as the carrier gas. Data acquisition and operation processing were conducted using ChemStation software (Agilent Technologies). Each SCFA was identified by its corresponding retention time and by spiking with standards in the same conditions. The amount of each SCFA was evaluated according to the peak area with the standard curve for quantitative analysis.

### Statistical analyses

All data were presented as means ± SD for 5–8 mice per each group. Statistical significance between groups was analyzed using GraphPad Prism software version 9 (GraphPad, San Diego, CA). To determine statistical significance between experimental groups, Student’s two-tailed t-test was used unless otherwise indicated. *P* values < 0.05 were considered as statistically significant. For data on body weight, the tissue weight/body weight ratio, the cecum weight/body weight ratio, the colon length, and plasma lipid analysis, significant differences were calculated using one-way analysis of variance (ANOVA) with Dunnett’s multiple comparison test was used with a *p* value less than 0.033.

## Results

### Transplantation of fecal microbiota from colitis mice did not drive inflammation or barrier disruption in the gut of GF recipient mice

To access the impact of colitis-associated altered microbiota and its metabolites on inter-organ crosstalk and host metabolism, we transplanted fecal microbiota from DSS-induced colitis mice to GF mice. The fecal-donor colitis mice were obtained by feeding male C57BL/6 mice with 3 cycles of 2% of DSS for 7 days and 7 days of drinking water between each cycle (Fig 1A), which was as described in our previous study [22]. The body weights of fecal-transplanted GF mice that received fecal microbiota from non-colitis or colitis mice, FMT-control or FMT-colitis groups, respectively, were not different from that of non-transplanted GF mice (Fig 1B). The enlarged cecum that is typical in GF mice [28] was reverted to normal size and weight in fecal-transplanted recipient mice groups, indicating that the transplanted microbial populations successfully colonized the gut of recipient mice (Fig 1C). There was also no significant difference in colon length shortening, one of the most frequently observed phenotypes in DSS-induced colitis mice, between FMT-control or FMT-colitis groups and their GF controls (Fig 1D). Stool consistency and rectal bleeding were additionally assessed, but there was also no significant difference in both stool consistency score and rectal bleeding score among the three groups, GF, FMT-control, and FMT-colitis. The mRNA expression levels of inflammatory cytokines in the colon showed mixed results between pro- and anti-inflammatory cytokines with increases in TNFα and IL-10, but no difference in IL-6, in FMT-colitis group compared with FMT-control group (Fig 1E). The mRNA expression of mucin and tight junction proteins including ZO-1, occludin, and claudins, in the colon and ileum also showed no differences between the two groups (Fig 1F and 1G), commensurate with no change in plasma LPS levels (Fig 1H). In addition, although we also measured the mRNA expression levels of pro-inflammatory cytokines in adipose tissues and the liver, the observed results showing the inflammatory state in the tissues did not indicate an overall trend of change in a certain direction (S1 Fig). These observations together indicated that the transplantation of fecal microbiota from DSS-induced colitis mice induced neither gut inflammation nor alterations of gut barrier integrity in the GF recipient mice.

### Transplantation of fecal microbiota from colitis mice reduced the weight of adipose tissue and caused its dysfunctions in GF recipient mice

**Fig 2:**
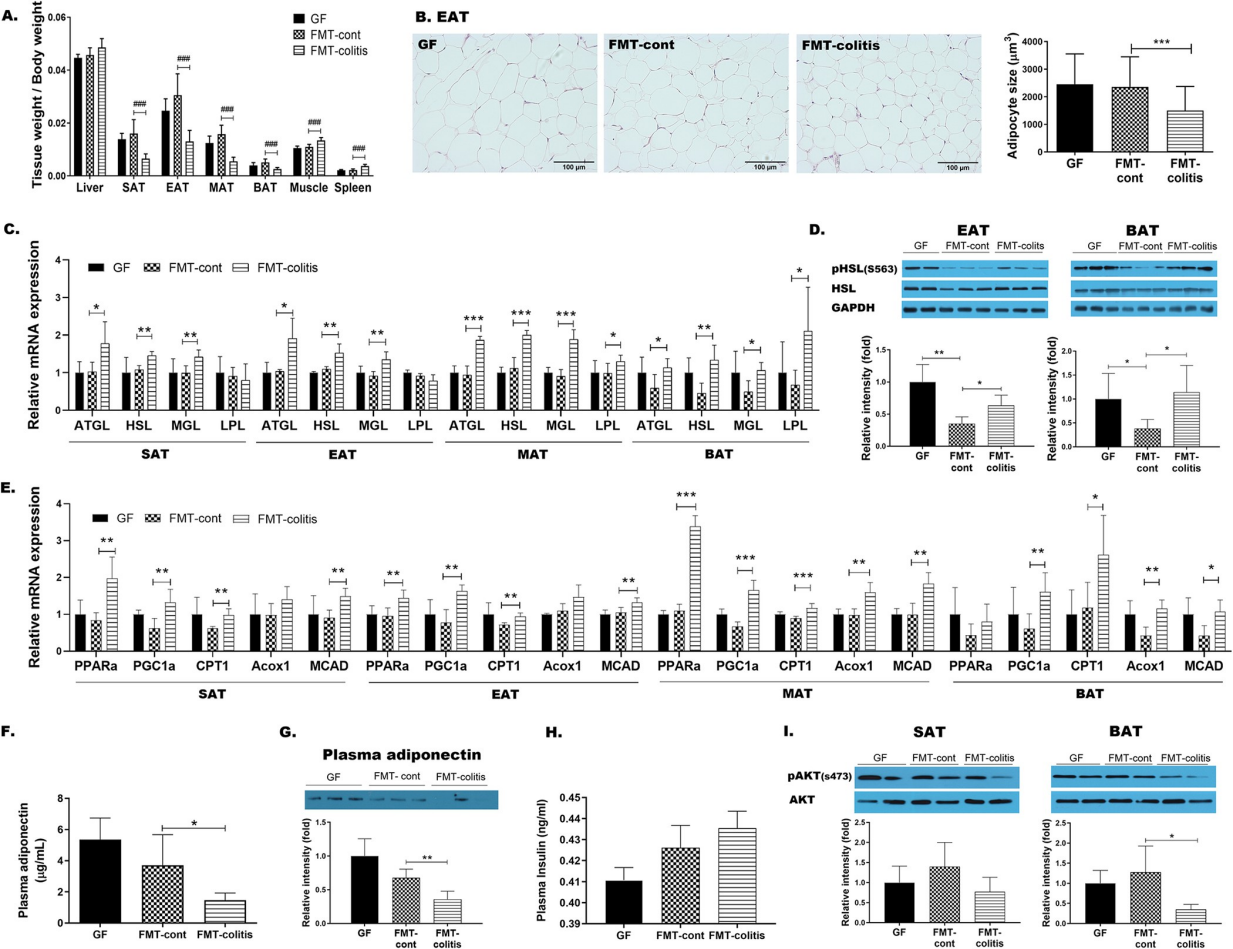
Transplantation of fecal microbiota from colitis mice reduced the weight of adipose tissue and caused its dysfunctions in GF recipient mice. (A) The ratio of tissue weight/body weight. In A, statistical significance was determined by ordinary one-way ANOVA with Dunnett’s multiple comparison test (^#^*p* < 0.033, ^##^*p* < 0.002, and ^###^*p* < 0.001). (B) Representative images (x200) of H&E stained adipose tissue sections and a graph presenting the size of adipocytes in EAT. (C) The gene expressions related lipolysis in MAT, EAT, SAT and BAT. (D) HSL phosphorylation (ser563) in EAT and BAT detected by SDS-PAGE-immunoblotting. (E) The gene expressions related fatty acid oxidation in MAT, EAT, SAT and BAT. Plasma adiponectin levels measured by (F) ELISA and (G) SDS-PAGE-immunoblotting. (H) Plasma insulin levels measured by ELISA. (I) Akt phosphorylation (ser473) in SAT and BAT detected by SDS-PAGE-immunoblotting. Data are presented as mean ± SD of each group (n = 5–7). In B-J, Student’s two-tailed t-test was used for analysis difference between FMT-control and FMT-colitis group (*p < 0.05, **p < 0.01, and ***p < 0.001).

Despite no significant differences in the body weight among the three groups, GF, FMT-control, and FMT-colitis group (Fig 1B), the FMT-colitis group mice showed a significant reduction in the weight of all adipose tissues including MAT, EAT, SAT, and BAT, compared to FMT-control group, which was not the case of other tissues (Fig 2A). In parallel to this reduction, the adipocyte sizes were also observed to be significantly reduced as representatively shown for EAT (Fig 2B) and SAT (S2A Fig). To examine why the weight of adipose tissues decreased in FMT-colitis group mice compared with FMT-control group, the changes in the expression of lipid-metabolic genes were assessed. The mRNA expression levels of the lipolytic enzymes such as ATGL, HSL, and MGL were significantly elevated in all tested adipose tissues (MAT, EAT, SAT, and BAT) of FMT-colitis group mice compared to FMT-control group (Fig 2C). The level of phosphorylated HSL were also significantly increased in FMT-colitis group mice compared to FMT-control group as representatively shown for EAT and BAT in Fig D. Additionally, the expression of genes related to fatty acid β-oxidation including PPARα, PGC1α, CPT1, and MCAD were also significantly increased in those tissue (Fig 2E). In contrast to the genes involved in lipolysis and β-oxidation, the expression of lipogenic genes did not show a certain trend of change; i.e., ChREBP in SAT, LXR and SCD1 in EAT, and ChREBP, SREBP1c and LXR in BAT were significantly increased, FAS in EAT and SREBP1c, LXR, ACC, and FAS in MAT were significantly decreased, but most of the related genes in SAT, EAT and BAT remained unchanged (S2B–S2E Fig). Taken together, our observations indicated that the reduced adiposity of adipose tissues in mice transplanted with fecal microbiota from colitis mice was due to the elevated lipolysis and fatty acid oxidation, but not de novo lipogenesis. Given that a reduced adipose tissue weight is normally accompanied with increased adiponectin production [29], it was unexpected result that the plasma level of adiponectin was remarkably lower in FMT-colitis group mice than that of FMT-control group (Fig 2F and 2G). Following these results showing abnormalities in adipose tissue, it was necessary to assess whether the insulin action in FMT-colitis group mice was normal. The plasma level of insulin was modestly, but not significantly, higher in FMT-colitis group mice than that of FMT-control group (Fig 2H) and the level of the phosphorylated Akt was also decreased in adipose tissues of FMT-colitis group mice as representatively shown for SAT and BAT in Fig 2I. However, the level of fasting blood glucose was not different between the two groups (S2F Fig), indicating that the transplantation with fecal microbiota from colitis mice caused an impairment of insulin action, but it was mild enough that it did not significantly raise blood glucose levels.

### Transplantation of fecal microbiota from colitis mice disrupted hepatic lipid metabolism in recipient GF mice

**Fig 3:**
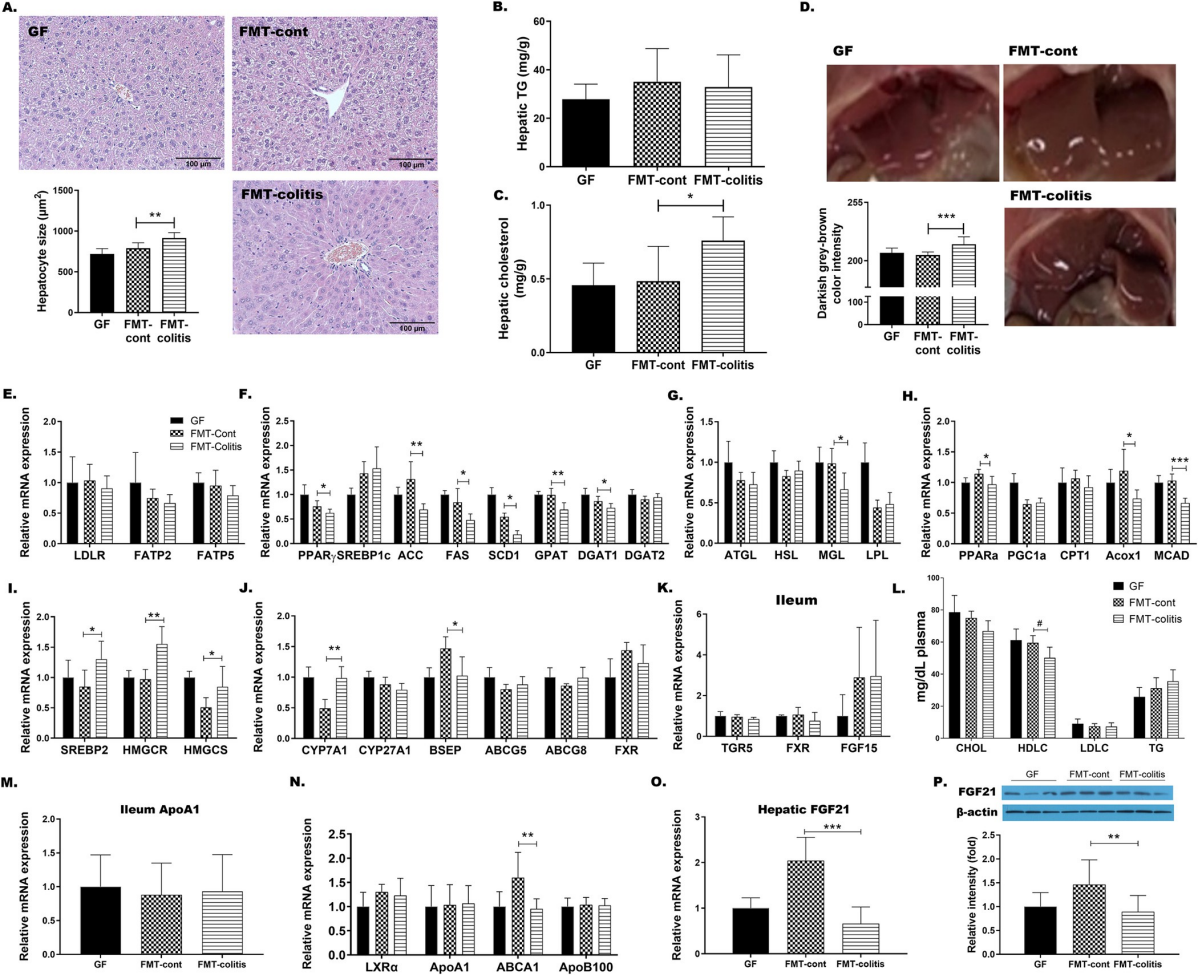
Transplantation of fecal microbiota from colitis mice disrupted hepatic lipid metabolism in recipient GF mice. (A) Representative images of H&E stained liver sections (left, x200) and a graph presenting the size of hepatocytes. (B-C) Hepatic TG and cholesterol content. (D) Representative images of the liver and a graph presenting the darkish grey-brown color intensity scaled from 0 (bright red-brown) to 255 (darkish grey-brown). The hepatic mRNA expression of genes related to (E) fatty acid uptake, (F) de novo lipogenesis, (G) lipolysis, (H) fatty acid oxidation, (I) cholesterol synthesis, and (J) the synthesis and excretion of bile acids. (K) The gene expression of TGR5, FXR, and FGF15 in distal ileum. (L) The plasma levels of cholesterol (CHOL), HDL-cholesterol (HDLC), LDL-cholesterol (LDLC), and triglyceride (TG). In L, statistical significance was determined by ordinary one-way ANOVA with Dunnett’s multiple comparison test (^#^*p* < 0.033, ^##^*p* < 0.002, and ^###^*p* < 0.001). (M) The gene expression of ApoA1 in distal ileum. (N) The expression of ApoB100 and genes related to reverse cholesterol transport. (O) Hepatic FGF21 gene expression and (P) plasma FGF21 levels. Data are presented as the mean ± SD of each group (n = 5–7). Statistically significant difference between FMT-control and FMT-colitis group was determined by Student’s two-tailed t-test (*p < 0.05, **p < 0.01, and ***p < 0.001).

The FMT-colitis group mice showed a modest, but not significant, increase in the liver weight compared with FMT-control group as shown in Fig 2A. With an aim to explore the effects of colitis-associated altered microbiota on the host metabolism of recipient mice, we specifically focused on this change. Having identified that the size of hepatocytes was significantly increased in FMT-colitis mice compared to FMT-control mice (Fig 3A), we first measured the hepatic content of triglyceride (TG). Interestingly, however, there was no difference between the two groups (Fig 3B), and instead, the hepatic cholesterol content was significantly higher in FMT-colitis group mice (Fig 3C). In addition, as shown in Fig 3D, it was histologically observed that the color of liver tissue of FMT-colitis group was more darkish grey-brown when compared with those of GF or FMT-control group mice, which were bright red-brown.

To elucidate the molecular mechanisms underlying these differences, we examined the changes in fat, cholesterol, and bile acid metabolism occurring in response to fecal microbiota transplantation. Consistent with unchanged level of TG content in the liver, the hepatic expressions of genes related to fatty acid uptake including LDLR, FATP2 and FATP5 (Fig 3E), and lipogenic genes including PPARɣ, SREBP1c, ACC, FAS, SCD1, GPAT, and DGATs were not changed or rather decreased in FMT-colitis mice compared to FMT controls (Fig 3F). The expression of lipolysis related genes including ATGL, HSL, MGL, and LPL, and β-oxidation related genes, including PPARα, PGC1α, CPT1, Acox1, and MCAD, also remained unchanged or rather decreased (Fig 3G and 3H). In contrast to these unchanged or reduced gene expressions in hepatic fat metabolism, the expression of genes involved in cholesterol synthesis, such as SREBP2, HMCGR and HMGCS was significantly upregulated in FMT-colitis group when compared with FMT-control group (Fig 3I). In addition, the expression of CYP7A1, the rate-limiting enzyme in classical bile synthesis pathway, was significantly increased in FMT-colitis group mice (Fig 3J). However, interestingly, the expression of genes involved in cholesterol excretion such as ABCG5, and ABCG8 was not changed, while the expression of BSEP, a major bile acid transporter, was significantly decreased in the liver of FMT-colitis mice compared to that of FMT-control group (Fig 3J). The ileal genes of bile acid receptors, TGR5 and FXR, and an enterohepatic signaling hormone FGF15 also showed no difference in expression levels (Fig 3K). These results together suggested that, in mice transplanted with fecal microbiota from colitis mice, the increased synthesis of cholesterol and bile acids, concomitant with decreased export of bile salts, resulted in the accumulation of cholesterol and bile salts in hepatocytes, consequently leading to the enlargement of hepatocytes and darkening of liver tissue color.

On the other hand, FMT-colitis group mice had a reduced plasma level of HDL-cholesterol compared to FMT-control group, as shown in Fig 3L. To examine the mechanism for this change, we tested the expression level of genes related to the reverse cholesterol transport (RCT). The gene expression of ApoA1, the major component of HDL-cholesterol, expression was not changed in both the liver and ileum (Fig 3M and 3N), however, the expression of ABCA1 that has important role in the formation of nascent HDL-cholesterol, was significantly decreased in the liver of FMT-colitis group when compared with FMT-control group (Fig 3N). In the case of LDL-cholesterol, there was no significant difference in its plasma level between the two groups, which was parallel to the result that showed no difference in the expression level of ApoB100, the major apolipoprotein of LDL (Fig 3N). Additionally, we also measured the hepatic expression of FGF21, a hepatokine that regulates glucose and lipid metabolism, and observed that its levels of mRNA and protein expression were significantly diminished in FMT-colitis group mice compared to FMT-control group (Fig 3O and 3P).

### GF mice transplanted with fecal microbiota from colitis mice displayed decreased levels of SCFAs and their receptors

**Fig 4:**
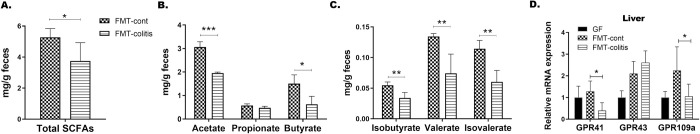
GF mice transplanted with fecal microbiota from colitis mice displayed decreased levels of SCFAs and their receptors. The fecal concentrations of (A) total SCFAs, (B) 3 major SCFAs, and (C) 3 minor SCFAs. (B) The mRNA expressions of SCFA receptors in the liver. Data are presented as the mean ± SD of each group (n = 5–7). Student’s two-tailed t-test was used for analysis difference between FMT-control and FMT-colitis (*p < 0.05, **p < 0.01, and ***p < 0.001).

Since it has been known that the FGF21 expression can be regulated by butyrate, which is one of the key metabolites of gut microbiota [16], our next experiment focused on SCFAs that were produced by fecal microbiota transplant. To confirm whether the physiological changes observed in GF mice transplanted with fecal microbiota from colitis mice was associated with microbiota-derived SCFAs, we measured the levels of fecal SCFAs and mRNA expression of their receptors in the target tissues. The total amount of fecal SCFAs was observed to be significantly decreased in FMT-colitis group mice compared with FMT-control group (Fig 4A). Also, we found that the fecal contents of acetate and butyrate, but not propionate, were significantly lower in FMT-colitis group mice (Fig 4B). Similarly, significantly reduced content of other SCFAs such as isobutyrate, valerate and isovalerate was also detected in the fecal samples of FMT-colitis group (Fig 4C).

On the other hand, in the expression of SCFA receptors such as GPR41, GPR43, and GPR109a, the expression pattern was observed to be different between the liver and adipose tissues. In the liver, the mRNA expression of GPR41 and GPR109a, but not GPR43, was significantly decreased in FMT-colitis compared to FMT-control group mice (Fig 4D), whereas, in adipose tissues, the expression of those receptors did not show any specific pattern (S3 Fig). The above data regarding SCFAs and their receptors suggested that the GF mice colonized with the microbiota sourced from colitis mice had reduced capacity of SCFA production and consequently lowered expression of SCFA receptors especially in the liver, which might contribute to the disturbance of hepatic lipid metabolism.

## Discussion

Numerous studies have shown that alterations in the gut microbiota and their metabolites are associated with the development and progression of major chronic inflammatory diseases including metabolic disorders and IBD [7]. Given that many epidemiological studies have reported the concomitance of metabolic abnormalities, especially NAFLD, among patients with IBD [20,21,29] and the gut microbiota dysbiosis is a common risk factor that is associated with both the two diseases [30–32], an understanding of molecular mechanism underlying how IBD-associated disruption of gut microbiota plays a role in the pathogenesis of NAFLD may provide promising candidate targets for therapeutic intervention for the comorbidity of the two diseases. In our previous study, it was found that DSS-induced colitis model mice concomitantly develop NAFLD phenotypes such as hepatic steatosis and dyslipidemia [22]. These colitis-associated metabolic abnormalities turned out to be due to hepatic inflammation and adipose tissue dysfunctions that led to disruptions of hepatic lipid and bile acid metabolism, resulting in excessive hepatic fat accumulation and abnormal lipid profiles. In this study, we hypothesized that colitis-associated alterations in gut microbiota and their-derived metabolites would disturb host energy metabolism and contribute to metabolic abnormalities.

Using GF mice transplanted with the fecal microbiota from donor mice with DSS-induced colitis, we determined whether the colitis-associated dysbiosis may cause the glucose- and/or lipid metabolism, and further tracked the key members of metabolic pathways responding to the dysbiotic microbiota and their metabolites. First, we compared the physiological phenotypes between the two groups of GF mice that received fecal transplants from DSS-induced colitis and non-colitis control mice (FMT-colitis vs. FMT-control group), and observed that the transplantation of colitis-associated dysbiotic microbiota did not change gut barrier integrity or induce systemic inflammation. However, interestingly, it was observed that the FMT-colitis group mice had some deleterious changes compared to FMT-control group; 1) wasting of all types of adipose tissue, 2) increased hepatocyte size, 3) darkened color of the hepatic tissue, and 4) reduced plasma level of HDL cholesterol. Although many recent studies have identified key roles of the gut microbiota in the pathogenesis of IBD [33,34], it remains unclear whether changes in the microbiota are a cause or a consequence of IBD [35]. Our observations suggest that the transplantation of the colitis-associated dysbiotic microbiota might not have an impact enough to cause gut barrier dysfunction and consequent endotoxemia leading to systemic chronic inflammation in recipient GF mice (Figs 1F–1H and S1). However, it was surprisingly found that the transplantation of dysbiotic microbiota caused some detrimental impacts especially on metabolic tissues including the liver and adipose tissue. The FMT-colitis group mice had a significant reduction in the weight of all types of adipose tissue compared to the FMT-control group, while the weight of liver tissue was not reduced but rather showed a tendency of modest increase (Fig 2A). Interestingly, these changes in adipose and liver tissue weights are consistent with those observed in DSS-induced colitis mice in our previous study [22]. In that study, we found that the extent of decrease in adipose tissue weight and increase in liver tissue weight was elevated in proportion to the severity of colitis induced by DSS. The results of the present study showing a decreased adipose tissue weight and an increased liver tissue weight in the GF mice that received the fecal microbiota from DSS-induced colitis mice suggest that those changes are possibly due to the colitis-associated microbiota dysbiosis. On the other hand, the muscle weight was significantly increased in FMT-colitis group mice (Fig 2A), which was inconsistent with the significant decrease observed in DSS-induced colitis mice [22]. The cause of the increase in muscle weight of GF mice transplanted with microbiota from colitis mice was unclear in the present study, and further study is required.

The examination focusing on the loss of adipose tissue caused by transplantation of the dysregulated microbiota from colitis mice provided some findings about metabolic changes occurred in adipose tissues. Levels of adiponectin, an adipokine that play a key role in maintaining metabolic health through promoting systemic insulin sensitivity and improving glucose and lipid metabolism [33], are higher in both non-obese and obese metabolically healthy individuals than metabolically unhealthy individuals [36]. Both unhealthy obesity and wasting diseases such as lipodystrophy and cachexia cause adipose tissue dysfunctions due to insufficient fat storage capacity, resulting in dyslipidemia and reduced adiponectin production, triggering the development of insulin resistance and further dysfunction of adipose tissue [37]. In this study, our data showed that there were significant reductions in weight and adipocyte size of WAT, accompanied with adipose tissue dysfunctions including reduced adiponectin production, reduced lipolysis, and increased fatty acid oxidation, leading to impaired fat storage, in FMT-colitis group mice compared to their controls (Figs 2A–2G and S2). This suggests that the transplantation of colitis-associated dysbiotic microbiota induces a fat wasting, which is similar to, but not the same as, that of lipodystrophy.

Over the past decade, deficiency of functional adipose tissue has been known to be associated with metabolic complications such as insulin resistance and dyslipidemia [38]. We also found in the present study that the GF mice that received colitis-associated dysbiotic microbiota displayed alterations in hepatic lipid metabolism along with adipose tissue dysfunctions. A modest, but not significant, increase in the liver weight and a significant increase in hepatocyte size in FMT-colitis group mice (Figs 2A and 3A) were observed to be due to a significant increase of cholesterol and bile acid synthesis (Fig 3C, 3I and 3J), but not of de novo lipogenesis (Fig 3B and 3F) or lipolysis (Fig 3G). However, inconsistent with the increased cholesterol synthesis in the liver, the level of plasma total cholesterol remained unchanged (Fig 3L), which was commensurate with an unchanged expression of ABCG5 and ABCG8 that are key to secreting cholesterol into the biliary lumen (Fig 3J).

On the other hand, while there was no difference in the plasma level of LDL-cholesterol, that of HDL-cholesterol was significantly reduced in FMT-colitis group (Fig 3L). These changes are also consistent with the results of our previous study, which showed an unchanged level of LDL-cholesterol and a reduced level of HDL-cholesterol, associated with the downregulation of genes related to RCT and cholesterol secretion in DSS-induced colitis mice [22]. These results obtained from our previous and present studies together suggest that colitis may be accompanied by low HDL-cholesterol dyslipidemia which is mediated by suppression of RCT and at least in part via a mechanism dependent on the colitis-associated dysbiosis. Although this hypo-HDL-cholesterolemia has not been known as a typical symptom of IBD, there have been several studies reporting that IBD patients had high prevalence of lower HDL-cholesterol [39,40]. Our findings in this study also indicate that patients with IBD are at an increased risk of developing low HDL-cholesterol dyslipidemia.

We also observed that the transplantation of colitis-associated dysbiotic microbiota caused a reduced production of FGF21 in the liver of GF recipient mice (Fig 3O and 3P). FGF21, a hormone that is mainly secreted by the liver and has important roles in regulating energy metabolism, induces adiponectin production from adipose tissue and coordinates adiponectin to improve insulin sensitivity and regulate glucose and lipid homeostasis [41,42]. This relationship between FGF21, adiponectin, and insulin might explain the changes in their levels observed in FMT-colitis group mice in this study, which were declines in FGF21 and adiponectin (Figs 2F, 2G, 3O and 3P) accompanied by elevated plasma insulin level (Fig 2H) and decreased insulin signaling in adipose tissue (Fig 2I). This could be interpreted that the transplantation of the dysregulated microbiota from colitis mice suppressed hepatic FGF21 production and subsequently decreased adiponectin secretion from adipose tissue, resulting in a reduced insulin sensitivity and a consequent induction of hyperinsulinemic compensation. However, it also should be noted that while there were a tendency of decreased insulin signaling in SAT and BAT and modestly elevated plasma insulin levels in FMT-colitis group mice, no significant elevation of fasting blood glucose indicative of insulin resistance was observed (S2F Fig), which indicated that the colonization of fecal microbiota from colitis mice in GF mice caused a disturbance in glucose metabolism, but not so severe that it can be called insulin resistance. Taken together, the transplantation of the colitis-associated dysbiotic microbiota was causally associated with impairment of FGF21-adiponectin axis, leading to metabolic abnormalities, including adipose tissue dysfunctions, dysregulated hepatic lipid metabolism, and reduced insulin sensitivity.

Our next question was how the colitis-associated dysbiotic microbiota could cause the disturbance of host energy metabolism. Accumulating evidence reveals that not only disrupted gut microbial composition but also metabolites derived from altered gut microbiota play important roles in the development of IBD [7]. However, there have been no reports made on the direct relationship showing the impact of a specific microbiota composition on host lipid and glucose metabolism in IBD, but instead, most reports have focused mainly on the effects of gut microbiota-derived metabolites, among which, particularly SCFAs are the most studied [9]. Many studies have demonstrated that the most abundant SCFAs such as acetate, propionate, and butyrate have important roles in maintaining intestinal barrier integrity and energy metabolism [43,44]. They exert beneficial effects on glucose and lipid metabolism and chronic inflammation through inhibition of histone deacetylation and/or binding to GPCRs such as GPR41, GPR43, and GPR109a [44], and reductions in their levels have been found to be associated with metabolic disorders or IBD [9]. In the same sense, the reduced SCFA synthetic capacity of gut microbiota has recently been shown to be a common hallmark of those diseases [9,45]. It has also been reported that gut dysbiosis-associated decrease in production of SCFAs, particularly butyrate, is correlated with the down-regulation of both adiponectin in adipose tissue and FGF21 in the liver [46–48]. In the present study, we found that GF mice colonized with the dysbiotic microbiota from colitis mice had reduced levels of fecal SCFAs as well as a down-regulation of hepatic expression of GPR41 and GPR109a receptors (Fig 4). Together with the results showing the reduced production of FGF21 and adiponectin, these findings suggest that the disrupted FGF21-adiponectin axis caused by the transplantation of colitis-associated dysbiotic microbiota is due to a lowered SCFA producing capacity of the microbiota. Unfortunately, in this study, we did not analyze the changes in the composition of the colonized microbiota of GF recipient mice, and thus, the inability to provide direct evidence for the reduced abundance of SCFA-producing bacteria is a limitation of this study.

**Fig 5:**
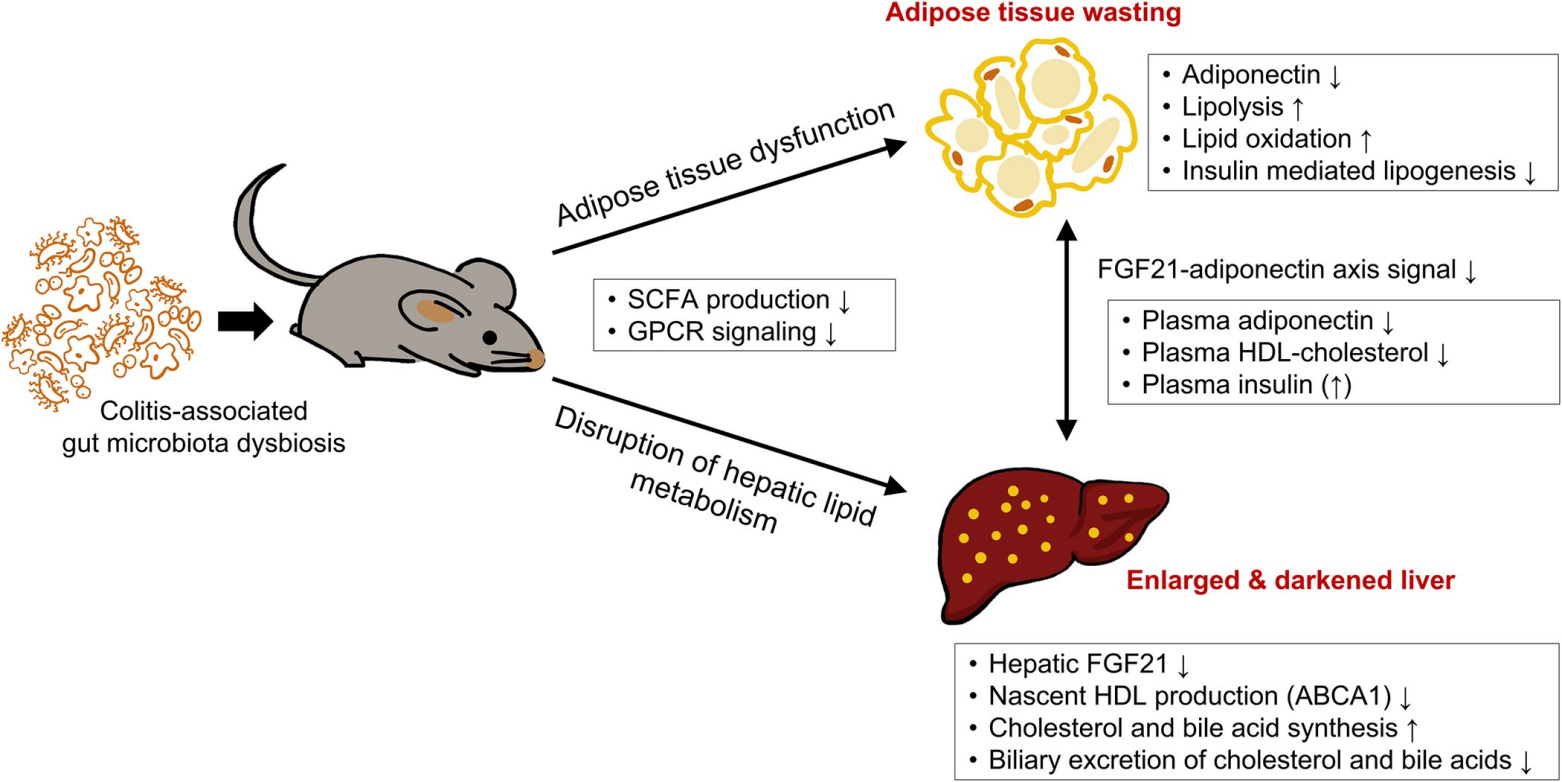
Possible mechanisms that explain how colitis-induced dysregulation of gut microbiota could lead to the disruption of host lipid metabolism.

Taken together, the metabolic abnormalities induced by colonization of GF mice with colitis-associated dysbiotic microbiota can be summarized as follows; (1) lipodystrophy-like wasting of adipose tissues, (2) hepatic lipid metabolic disturbance with an increase in synthesis of cholesterol and bile acids and a decrease in their biliary excretion, leading to increased hepatocyte size and liver weight, (3) hypo-HDL-cholesterolemia, and (4) FGF21-adiponectin axis impairment and hyperinsulinemia, each of which is, at least in part, due to the low SCFA-producing capacity of the colonized microbiota. These findings suggest potential mechanisms that explain how colitis-associated gut microbiota dysbiosis may contribute to the development of metabolic dysfunctions, as shown in Fig 5.

Our previous study has reported that DSS-induced colitis is associated with adipose tissue dysfunction and disrupted hepatic lipid metabolism leading to hepatosteatosis and dyslipidemia in mice, suggesting the molecular mechanisms underlying the comorbidity between IBD and metabolic disorders [22]. We conclude in the present study that the colitis-induced gut dysbiosis plays an important role in the pathogenesis of metabolic disorders in patients with IBD and it should be considered as a crucial link in the mechanism of the association between the two diseases. This understanding of possible molecular and pathophysiological links may have potential to be applied to clinical practice where their targeted therapy could improve the efficacy of treatment of IBD patients with comorbid metabolic disorders or vice versa.

## Supporting information

S1 Fig The gene expression of pro-inflammatory cytokines in adipose tissues and the liver. (A-D) The gene expressions of TNFα, IL-6, IL-1β, and MCP-1 in adipose tissues and the liver.
(TIF)

S2 Fig H&E stained SAT sections, the gene expression related *de novo* lipogenesi*s* in adipose tissues, and fasting blood glucose. (A) Representative images (x200) of H&E stained SAT sections and a graph presenting the size of adipocytes in SAT. (B-E) The gene expressions related *de novo* lipogenesis in adipose tissues. (F) Fasting blood glucose.
(TIF)

S3 Fig The gene expression of SCFAs receptors in adipose tissues. (A-D) The gene expressions of GPR41, GPR43, GPR109a in adipose tissues.
(TIF)

S1 Table Primer sequences for real-time PCR. (DOCX)

S1 Data (ZIP)

S1 Raw images (PDF)
