# Supplementary figures and images for: Disturbance of lipid metabolism in germ-free mice transplanted with gut microbiota of DSS-induced colitis mice
Source: PLoS One. 2023 Feb 3;18(2):e0280850. doi: 10.1371/journal.pone.0280850 (PMC9897547; doi:10.1371/journal.pone.0280850)

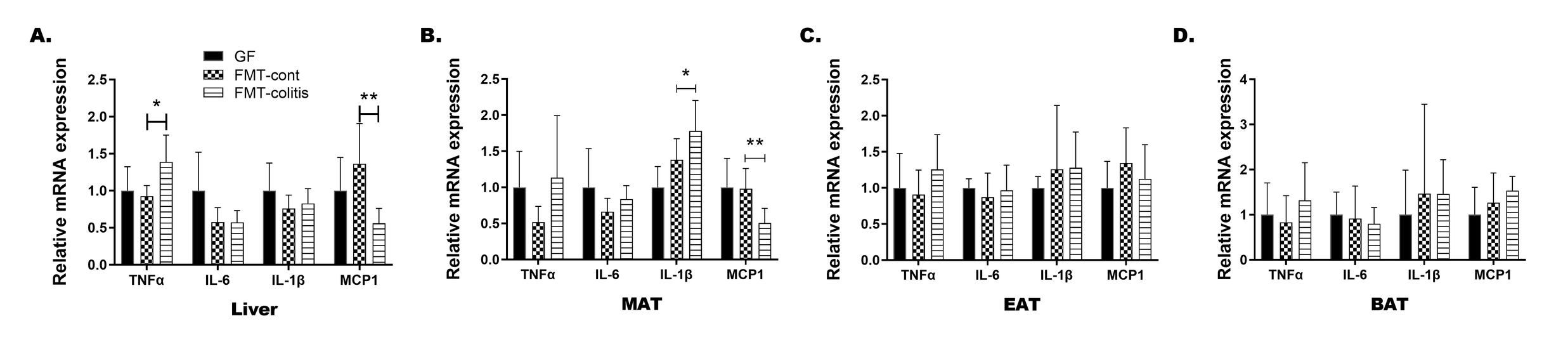

Supplement: S1 Fig — (A-D) The gene expressions of TNFα, IL-6, IL-1β, and MCP-1 in adipose tissues and the liver. (TIF) [file pone.0280850.s001.tif]

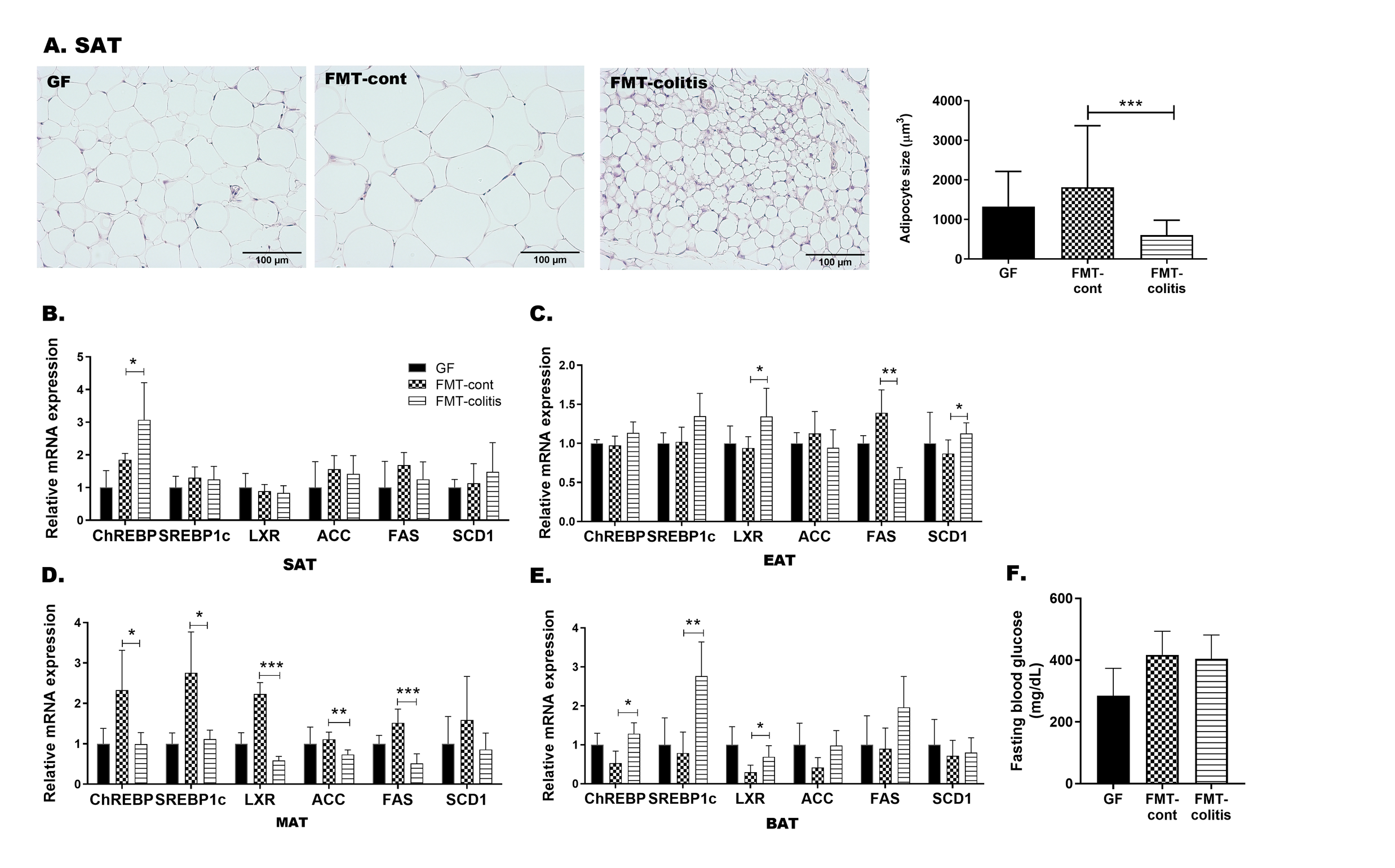

Supplement: S2 Fig — (A) Representative images (x200) of H&E stained SAT sections and a graph presenting the size of adipocytes in SAT. (B-E) The gene expressions related de novo lipogenesis in adipose tissues. (F) Fasting blood glucose. (TIF) [file pone.0280850.s002.tif]

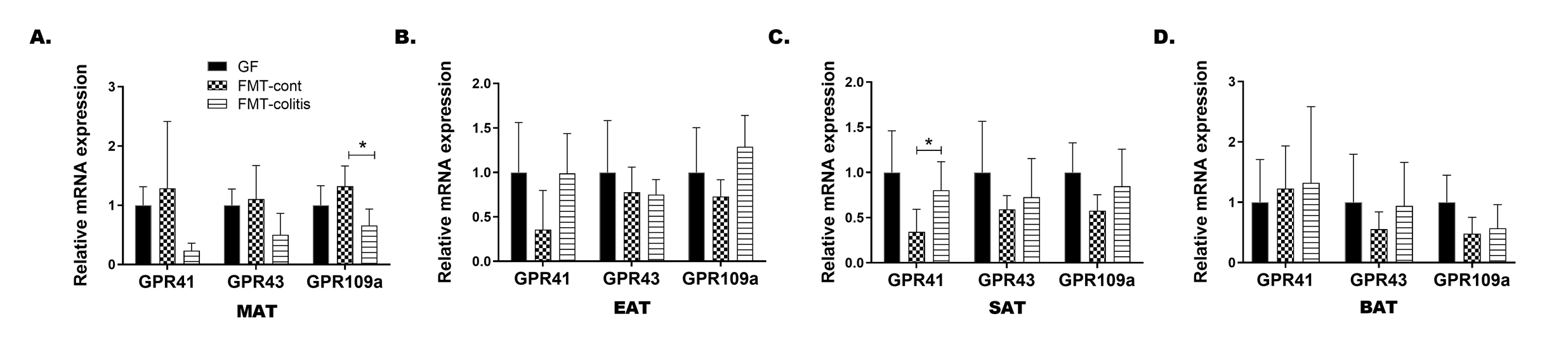

Supplement: S3 Fig — (A-D) The gene expressions of GPR41, GPR43, GPR109a in adipose tissues. (TIF) [file pone.0280850.s003.tif]
