# Supplementary material for: Disturbance of lipid metabolism in germ-free mice transplanted with gut microbiota of DSS-induced colitis mice
Source: PLoS One. 2023 Feb 3;18(2):e0280850. doi: 10.1371/journal.pone.0280850 (PMC9897547; doi:10.1371/journal.pone.0280850)
Supplement: S1 Table — (DOCX) [file pone.0280850.s004.docx]

**S1 Table. Primer sequences for real-time PCR**

| **Gene** | | **Primer sequence** |
| --- | --- | --- |
| Arbp | Forward | TCACTGTGCCAGCTCAGAAC |
|  | Reverse | AATTTCAATGGTGCCTCTGG |
| ABCA1 | Forward | GCGACCATGAAAGTGACACG |
|  | Reverse | CAGCACATAGGTCAGCTCGT |
| ABCG5 | Forward | CCTGCTGAGGCGAGTAACAA |
|  | Reverse | GGACGCGGAGAAGGTAGAAA |
| ABCG8 | Forward | GAAAAGAACCAGCGGGGAGA |
|  | Reverse | GCCTGGGATTTTGCCTACCT |
| ACC | Forward | TGACAGACTGATCGCAGAGAAAG |
|  | Reverse | TGGAGAGCCCCACACACA |
| Acox1 | Forward | GTGCAGCTCAGAGTCTGTCCAA |
|  | Reverse | TACTGCTGCGTCTGAAAATCCA |
| ApoA1 | Forward | CAGAGACTATGTGTCCCAGTTTGAATCCT |
|  | Reverse | TATCCCAGAAGTCCCGAGTCAATGGGCCC |
| ApoB100 | Forward | AGGTACGAACTCAAGCTGGC |
|  | Reverse | GAGCAGAGATGATGCCCCTC |
| ATGL | Forward | GACAGCTCCACCAACATCCA |
|  | Reverse | GCAAAGGGTTGGGTTGGTTC |
| β-actin | Forward | GGCACCACACYTTCTACAATG |
|  | Reverse | GGGGTGTTGAAGGTCTCAAAC |
| BSEP | Forward | CTGCCAAGGATGCTAATGCA |
|  | Reverse | CGATGGCTACCCTTTGCTTCT |
| ChREBP | Forward | CTGGGGACCTAAACAGGAGC |
|  | Reverse | GAAGCCACCCTATAGCTCCC |
| Claudin2 | Forward | GGCTGTTAGGCACATCCAT |
|  | Reverse | TGGCACCAACATAGGAACTC |
| Claudin3 | Forward | AAGCCGAATGGACAAAGAA |
|  | Reverse | CTGGCAAGTAGCTGCAGTG |
| CPT1 | Forward | TGAGTGGCGTCCTCTTTGG |
|  | Reverse | TCAGCGAGTAGCGCATAGTCA |
| CYP27A1 | Forward | AGTGATGAGACAGGAGGGCA |
|  | Reverse | TCCTTGTGCGATGAAGATCCC |
| CYP7A1 | Forward | GGTCCTCCAGCAGAGAGCTA |
|  | Reverse | AGGAAGGAAGCATAGCGTACC |
| DGAT1 | Forward | TCAGATTGAGAAGCGCCTGG |
|  | Reverse | ACGGAACCCACTGGAGTGAT |
| DGAT2 | Forward | GCCGATGGGTCCAGAAGAAGTT |
|  | Reverse | CTCCAGCTTGGGGACAGTGATG |
| FAS | Forward | CTGGACTCGCTCATGGGTG |
|  | Reverse | CATTTCCTGAAGTTTCCGCAG |
| FGF15 | Forward | AGTACCTGTACTCCGCTGGT |
|  | Reverse | ACGTCCTTGATGGCAATCGT |
| FGF21 | Forward | CGAGGCTGAAAAGATGGCCT |
|  | Reverse | GCGGCAGAAGAGAGCTATAACA |
| FXR | Forward | TGAGACTGGGTACCAGGGAG |
|  | Reverse | CAACACACAGCTCATCCCCT |
| FATP2 | Forward | TCCTCCAAGATGTGCGGTACT |
|  | Reverse | TAGGTGAGCGTCTCGTCTCG |
| FATP5 | Forward | GACTTTTGATGGGCAGAAGC |
|  | Reverse | GGGCCTTGTTGTCCAGTATG |
| GAPDH | Forward | TGTGTCCGTCGTGGATCTGA |
|  | Reverse | CCTGCTTCACCACCTTCTTGAT |
| GPAT | Forward | CAGACACAGGCAGGGAATCC |
|  | Reverse | GCCTAGGTCGAAATCGCGAG |
| GPR109a | Forward | CGAGATGTGGAAGCCAGATAA |
|  | Reverse | AGTATTCCAGGGGCGCTAAT |
| GPR41 | Forward | TTTCTGAGCGTGGCCTATCC |
|  | Reverse | ACACTACAGTGAGCCGATGC |
| GPR43 | Forward | GTGCTCCGCTGATTTTCATA |
|  | Reverse | ACGGGTACCAAGACATCTCC |
| HMGCR | Forward | CTTGTGGAATGCCTTGTGATTG |
|  | Reverse | AGCCGAAGCAGCACATGAT |
| HMGCS | Forward | CCAAGACTCCCTGCAACCTC |
|  | Reverse | CCAACCGTTTCCATACCCCA |
| HSL | Forward | CGGAACTAAGTGGACGCAAGC |
|  | Reverse | TCAGACACACTCCTGCGCATA |
| IL-10 | Forward | AGGCGCTGTCATCGATTTCT |
|  | Reverse | ATGGCCTTGTAGACACCTTGG |
| IL-4 | Forward | GGCATTTTGAACGAGGTCACA |
|  | Reverse | GACGTTTGGCACATCCATCTC |
| LDLR | Forward | CTGTGATCCGAGTGAGGACG |
|  | Reverse | AGTCTTCTGCTGCAACTCCG |
| LPL | Forward | GATCCGAGTGAAAGCCGGAG |
|  | Reverse | TGTTTGTCCAGTGTCAGCCAG |
| LXRα | Forward | GTGGGGAAGCTACCTCGTG |
|  | Reverse | GTAGGAAAAGGCCAAGGGGAG |
| MCAD | Forward | AACTAAACATGGGCCAGCGA |
|  | Reverse | GAAACCTGCTCCTTCACCGA |
| MGL | Forward | AAACAGACTTGTGCCCGTCA |
|  | Reverse | CGACCGCTTAGGGAAGGAAA |
| MUC2 | Forward | GGGAGGGTGGAAGTGGCATTGT |
|  | Reverse | TGCTGGGGTTTTTGTGAATCTC |
| Occludin | Forward | ATGTCCGGCCGATGCTCTC |
|  | Reverse | TTTGGCTGCTCTTGGGTCTGTAT |
| PGC1α | Forward | CCTGAAGCCGGGAGAGAATG |
|  | Reverse | TAGCCAGCAGAGACTGTGGA |
| PPARα | Forward | GTACGGTGTGTATGAAGCCATCTT |
|  | Reverse | GCCGTACGCGATCAGCAT |
| PPARγ | Forward | AGTGGAGACCGCCCAGG |
|  | Reverse | GCAGCAGGTTGTCTTGGATGT |
| SCD1 | Forward | TCAACTTCACCACGTTCTTCA |
|  | Reverse | CTCCCGTCTCCAGTTCTCTT |
| SPREBP2 | Forward | TGTGGAGCAGTCTCAACGTC |
|  | Reverse | GCTTTTGCCAGAGTGCTGTC |
| SR-B1 | Forward | GTGCCCATCATCTGCCAACT |
|  | Reverse | TGGTGACATCAGGGACTCAGA |
| SREBP1c | Forward | AGCAGCCCCTAGAACAAACAC |
|  | Reverse | CAGCAGTGAGTCTGCCTTGAT |
| TGR5 | Forward | CTTCTCTCTGTCCGCGTGTT |
|  | Reverse | GCCAGGGTTGAGGGTACATC |
| TNFα | Forward | GAGGCTCCAGTGAATTCGGA |
|  | Reverse | CACAAGATGCTGGGACAGTGA |
| ZO-1 | Forward | TTTTTGACAGGGGGAGTGG |
|  | Reverse | TGCTGCAGAGGTCAAAGTTCAAG |
