# Supplementary material for: Disturbance of lipid metabolism in germ-free mice transplanted with gut microbiota of DSS-induced colitis mice
Source: PLoS One. 2023 Feb 3;18(2):e0280850. doi: 10.1371/journal.pone.0280850 (PMC9897547; doi:10.1371/journal.pone.0280850)
Supplement: S1 Raw images — (PDF) [file pone.0280850.s006.pdf]

In Fig 2D

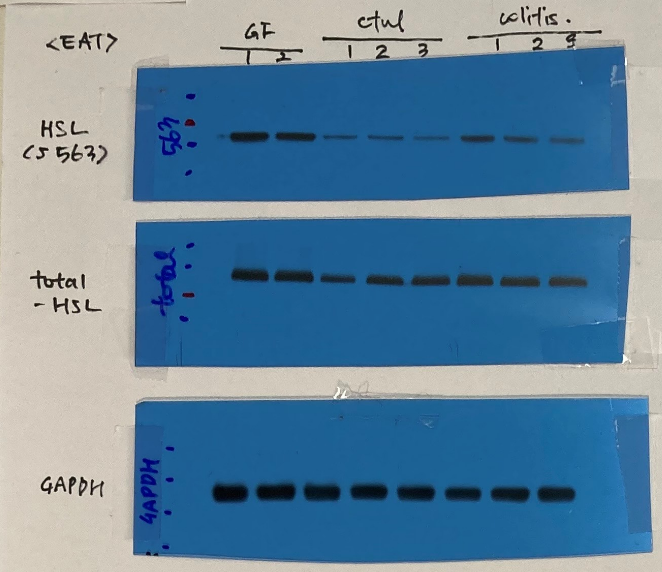

In Fig 2D

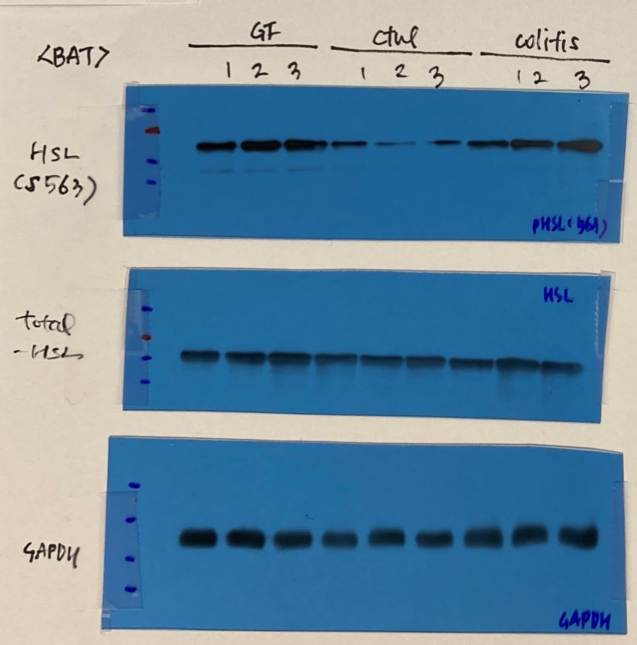

In Fig 2G

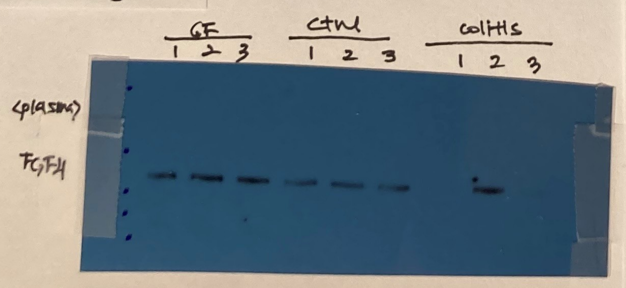

In Fig 3P

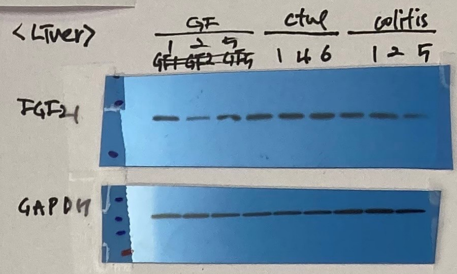

In Fig 2I

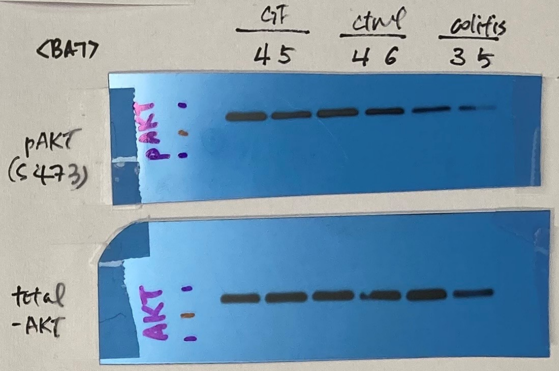

In Fig 2I

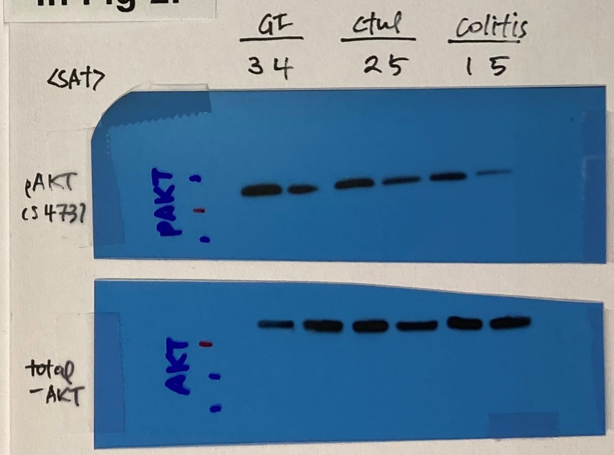

The way we obtained the blot images is as follows;

1) After electroblotting, we cut the blot membrane itself into strips. Referring to the position of the size marker, the region corresponding to the position of the protein of interest was cut out.

2) In a dark room, we pre-cut the X-ray film to approximately the size of the membrane strip (usually large enough to cover the membrane strip).

3) We placed the sheet of pre-cut film on top of the wrapped membrane in a cassette for exposure.

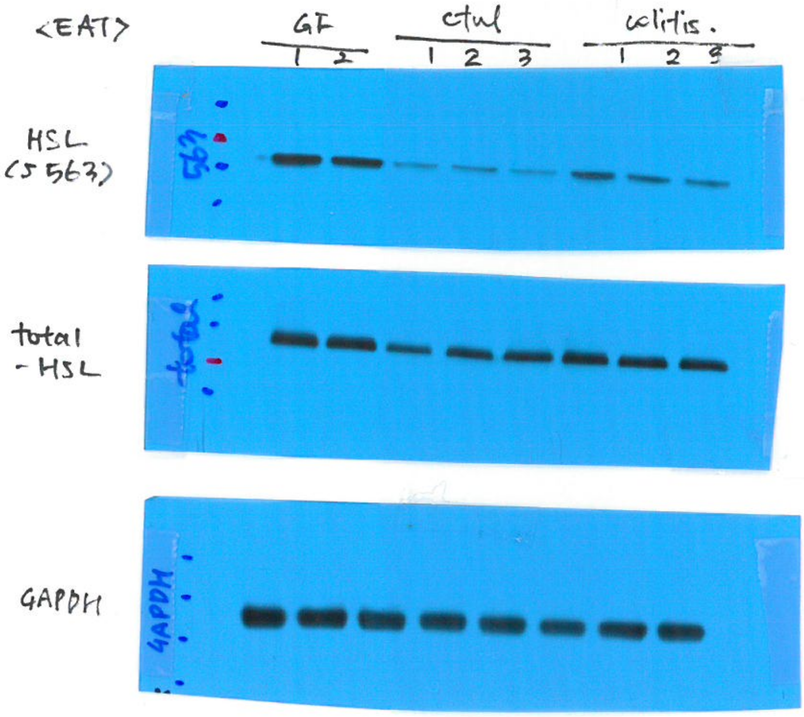
<EAT>
GF
1 2
ctrl
1 2 3
colitis.
1 2 3
HSL (S563)
total -HSL
GAPDH

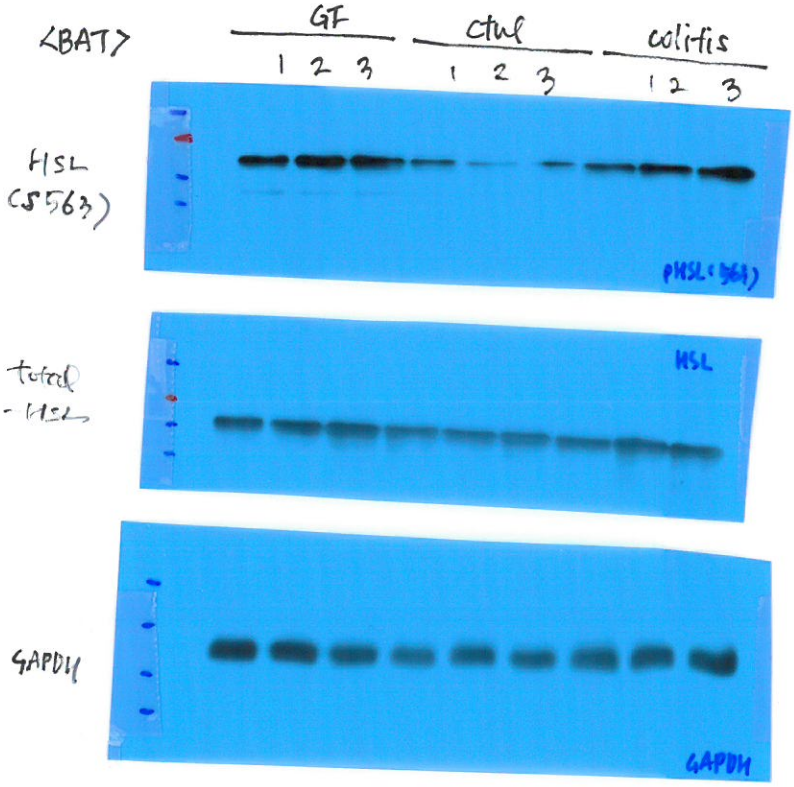
<BAT>
GF
1 2 3
ctrl
1 2 3
colitis
1 2 3
HSL (S563)
pHSL(563)
total -HSL
HSL
GAPDH
GAPDH

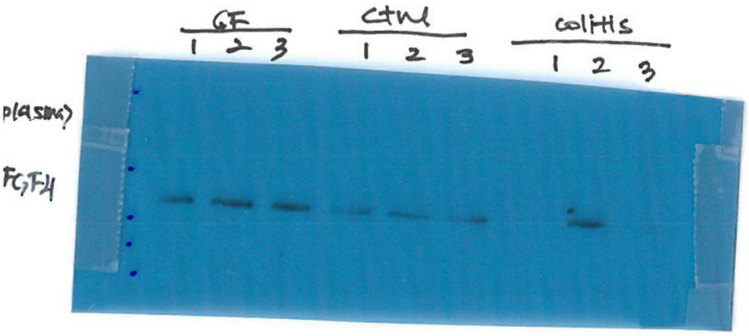
GF
1 2 3
ctrl
1 2 3
colitis
1 2 3
plasma
FGF21

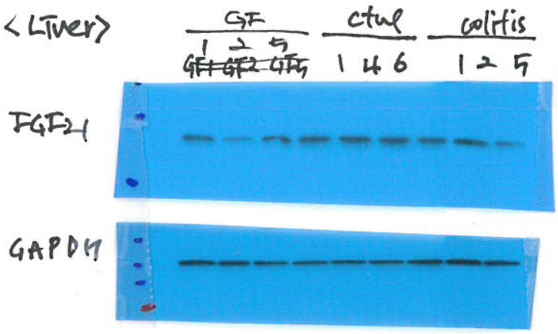
<Liver>
GF
1 2 5
~~GF1 GF2 GF5~~
ctrl
1 4 6
colitis
1 2 5
FGF21
GAPDH

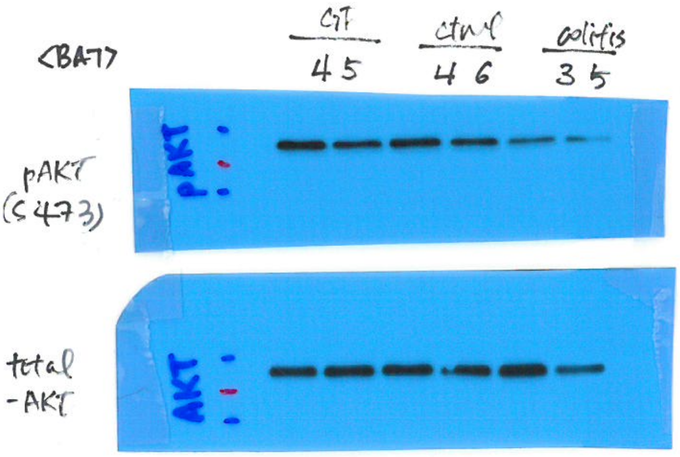
<BAT>
GF
4 5
ctrl
4 6
colitis
3 5
pAKT
(S473)
pAKT
total
-AKT
AKT

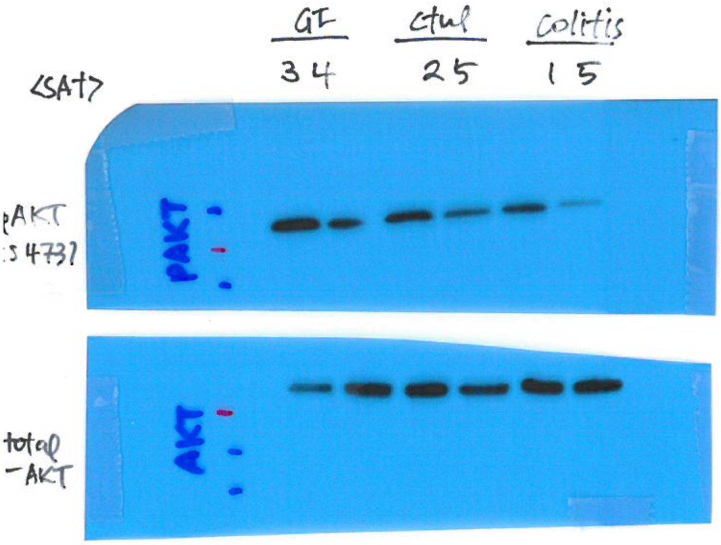
<SAT>
GF
3 4
ctrl
2 5
colitis
1 5
pAKT
(S473)
pAKT
total
-AKT
AKT
